# Robust transcriptional signatures for low-input RNA samples based on relative expression orderings

**DOI:** 10.1186/s12864-017-4280-7

**Published:** 2017-11-28

**Authors:** Huaping Liu, Yawei Li, Jun He, Qingzhou Guan, Rou Chen, Haidan Yan, Weicheng Zheng, Kai Song, Hao Cai, You Guo, Xianlong Wang, Zheng Guo

**Affiliations:** 1 Department of Bioinformatics, Key Laboratory of Ministry of Education for Gastrointestinal Cancer, School of Basic Medical Sciences, Fujian Medical University, Fuzhou, 350122 China; 2 Fujian Key Laboratory of Tumor Microbiology, Fujian Medical University, Fuzhou, 350122 China; 3 Department of Systems Biology, College of Bioinformatics Science and Technology, Harbin Medical University, Harbin, 150086 China; 4 Key Laboratory of Medical bioinformatics, Fujian Province, China

**Keywords:** Low-input RNA samples - amplification artificial signals - relative expression orderings - transcriptional signatures

## Abstract

**Background:**

It is often difficult to obtain sufficient quantity of RNA molecules for gene expression profiling under many practical situations. Amplification from low-input samples may induce artificial signals.

**Results:**

We compared the expression measurements of low-input mRNA samples, from 25 pg to 1000 pg mRNA, which were amplified and profiled by Smart-seq, DP-seq and CEL-seq techniques using the Illumina HiSeq 2000 platform, with those of the paired high-input (50 ng) mRNA samples. Even with 1000 pg mRNA input, we found that thousands of genes had at least 2 folds-change of expression levels in the low-input samples compared with the corresponding paired high-input samples. Consequently, a transcriptional signature based on quantitative expression values and determined from high-input RNA samples cannot be applied to low-input samples, and vice versa. In contrast, the within-sample relative expression orderings (REOs) of approximately 90% of all the gene pairs in the high-input samples were maintained in the paired low-input samples with 1000 pg input mRNA molecules. Similar results were observed in the low-input total RNA samples amplified and profiled by the Whole-Genome DASL technique using the Illumina HumanRef-8 v3.0 platform. As a proof of principle, we developed REOs-based signatures from high-input RNA samples for discriminating cancer tissues and showed that they can be robustly applied to low-input RNA samples.

**Conclusions:**

REOs-based signatures determined from the high-input RNA samples can be robustly applied to samples profiled with the low-input RNA samples, as low as the 1000 pg and 250 pg input samples but no longer stable in samples with less than 250 pg RNA input to a certain degree.

**Electronic supplementary material:**

The online version of this article (10.1186/s12864-017-4280-7) contains supplementary material, which is available to authorized users.

## Background

Gene expression profiling based on microarray and RNA sequencing techniques allows us to comprehensively characterize RNA transcripts present in a biological sample. However, it is often difficult to obtain sufficient quantity of RNA molecules for gene expression profiling under many practical situations. For example, minimally invasive tissue biopsy techniques, such as fine needle aspiration cytology, core needle biopsy and gastrointestinal endoscopy, are widely used clinically but minimum samples are extracted [1–3]. For another example, in formalin-fixed paraffin-embedded tissue samples with abundant clinical information, the amount of RNA is often limited due to partial RNA degradation [4, 5]. In the studies of rare cell population, single cell [6, 7] or the samples taken with the laser capture microdissection [8] technique, the amount of RNA molecules is also extremely low.

It is critical to overcome this challenge to leverage the power of low-input sampling techniques for biomedical applications. For this type of samples, multiple rounds of pre-amplification are necessary prior to the measurements of gene expression levels. Thus, a number of low-input RNA amplification techniques prior sequencing have been developed using PCR or in vitro transcription (IVT) to synthesize enough cDNA or cRNA, such as Smart-seq (switching mechanism at 5′-end of the RNA transcript) [9], DP-seq (primer-based RNA-sequencing strategy) [10] and CEL-seq (cell expression by linear amplification and sequencing) [11]. However, current low-input amplification techniques usually bring a large bias due to the inherent defects in the amplification principles [12]. For example, CEL-seq incorporating IVT can result in 3′ biases due to two rounds of reverse transcription before the linear amplification [11, 13]. Smart-Seq, using PCR to synthesize cDNA, is a nonlinear amplification process, and its efficiency is sequence-dependent [9, 13]; a long transcript may be truncated due to inefficient cDNA synthesis during the amplification process [14, 15]. It has been reported that the amplification bias always exists in lowly expressed genes and genes with abundant CG and long length [16–18]. As a result, it is uncertain whether the expression values measured after the amplification can represent the real gene expression levels or not.

Several studies attempted to prove that gene expression profiling can be performed on low-input RNA samples like high-input RNA samples by showing that the gene expression profiles of low-input RNA samples are significantly correlated with those of the matched high-input RNA samples [19–21]. However, a high correlation between two measurements does not guarantee that the two measurements are congruent, which brings uncertainty to the application of most current disease signatures based on risk scores which are calculated using the measurement values of the signature genes. Therefore, for a transcriptional signature based on the quantitative expression levels, the risk score thresholds determined from high-input RNA samples may be not applicable to low-input RNA samples, and vice versa. It has been reported that quantitative transcriptional signatures lack robustness for clinical applications due to measurement batch effects [22], variations of the tumor epithelial cell proportions in tissues sampled from different sites of a tumor [23, 24] and partial RNA degradation during sample preparation [25, 26]. Another type of disease signature is based on the within-sample relative expression orderings (REOs) of gene pairs [27, 28], which have been identified for predicting the prognosis of colorectal cancer [29], non-small cell lung cancer [30], ER+ breast cancer [31] and other cancers [32, 33]. These REOs-based signatures are robust against various measurement biases introduced by experimental batch effects and platform differences [34], partial RNA degradation [26] and uncertain sampling sites within the same cancer tissue [24]. Thus, we hypothesized that the REOs of gene pairs within individual samples, especially those with large rank differences, might also be robust against the biases introduced by the RNA amplification procedures.

Through comparing gene expression profiles between the samples with low-input mRNA, ranging from 25 pg to 1000 pg mRNA profiled by the Illumina HiSeq 2000 platform, and their paired high-input 50 ng mRNA samples, we found that there were thousands of genes with at least 2 folds-change (FC) in their expression values even when the input mRNA was 1000 pg. We evaluated the proportions of REOs of gene pairs in the high-input RNA samples maintained in the low-input RNA samples, and found that the proportions were approximately 90% even when the input mRNA was as low as 1000 pg and the input total RNA samples was as low as 250 pg, which suggests that REOs measured in the low-input samples were robust against amplification. Similar results were also found in the low input total RNA ranging from 10 pg to 1000 pg profiled by the Illumina HumanRef-8 v3.0 platform compared with the 100 ng input total RNA samples. As a case study to demonstrate the robustness of REOs-based signatures, we developed REOs-based signatures from high-input RNA samples for discriminating cancer tissues and showed that they can be robustly applied to low-input RNA samples.

## Results

### Large amplification bias of low-input RNA samples

**Fig. 1:**
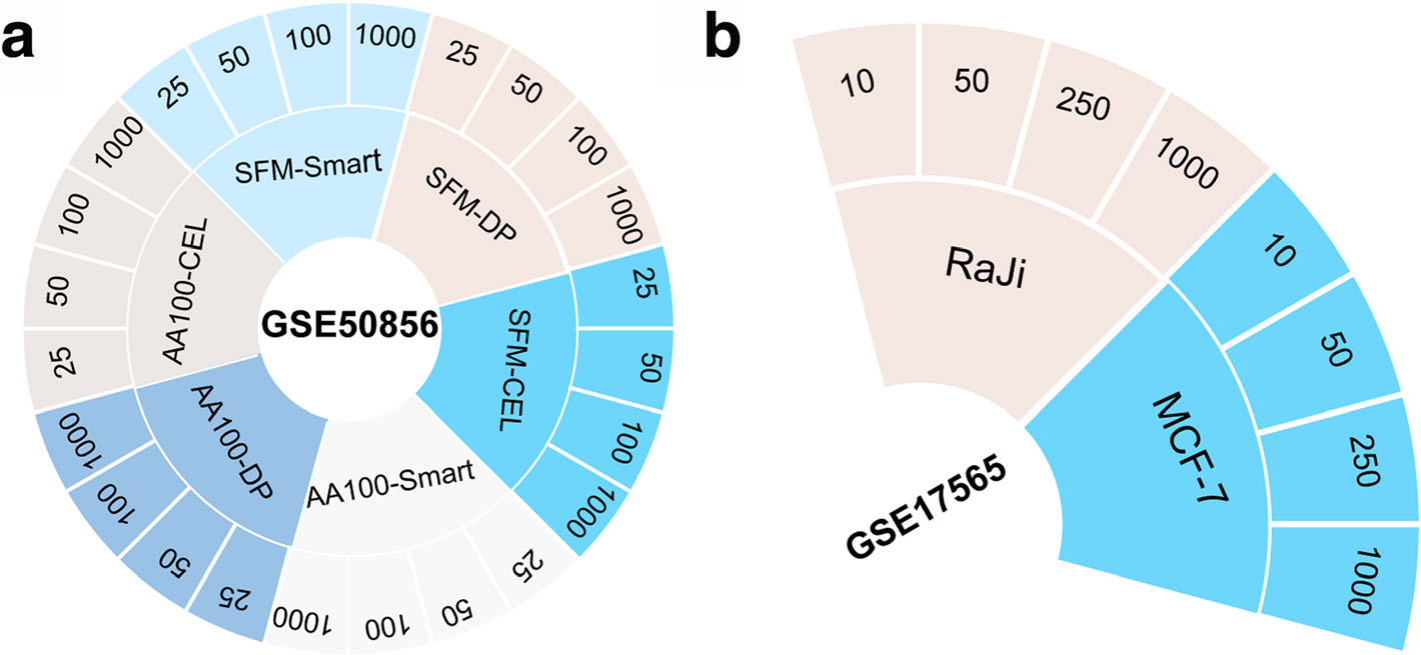
Datasets in this study. **a** The GSE50856 dataset was divided into 6 groups: SFM-Smart, SFM-DP, SFM-CEL, AA100-Smart, AA100-DP and AA100-CEL. Each group had four low input mRNA levels, 1000 pg, 100 pg, 50 pg and 25 pg, and each level had two technical replicates. **b** The GSE17565 dataset was divided into 2 groups: Raji and MCF-7. Each group had four low input total RNA levels, 1000 pg, 250 pg, 50 pg and 10 pg, and each level had two technical replicates

Based on two datasets (GSE50856 and GSE17565, see Fig. 1) measured by Illumina HiSeq 2000 and Illumina HumanRef-8 v3.0 platforms, respectively, we evaluated the amplification fidelity of low-input RNA samples amplified by several techniques through comparison with the corresponding high-input RNA samples using the FC values.

**Fig. 2:**
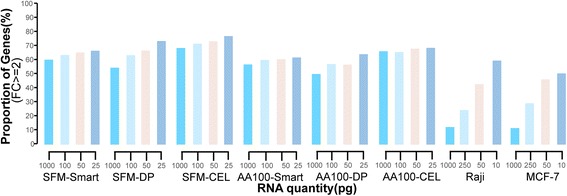
Amplification bias. Proportion of genes with at least 2 folds-change of expression values

In the SFM-Smart group of dataset GSE50856, there were respectively 60.56, 64.00, 65.76 and 66.95% of genes with a FC value larger than or equal to 2 in the expression values between 1000 pg, 100 pg, 50 pg and 25 pg mRNA samples compared with the paired high-input samples. As the amount of RNA in the diluted low input samples decreased, the percentage of genes with at least 2 FC increased. Similar results were also observed in the other five groups of dataset GSE50856 (Fig. 2). In the SFM-smart data, the coefficient of variation (CV) of FCs increased from 0.18 to 0.33 as the quantity of the input RNA decreased from 1000 pg to 25 pg (Additional file 1: Figure 1Sa). Similar results were observed in the data for SFM-DP, SFM-CEL, AA100-Smart, AA100-DP, AA100-CEL, Raji and MCF-7 (Additional file 1: Figure 1Sa, b and c). Thus, a large amplification bias exists for the three amplification techniques even the amplification begins from 1000 pg mRNA input.

For the Raji group of dataset GSE17565, there were 12.02, 23.79, 41.73, 57.84% of genes with a FC value larger than or equal to 2 in the expression values, respectively, in the 1000 pg, 250 pg, 50 pg, 10 pg samples compared with the paired high-input samples. Similar results were also observed for the MCF-7 group (Fig. 2). Obviously, the amplification procedure has a profound negative impact on the measurements of gene expression levels of the low-input samples.

### Robustness of REOs against amplification bias

Using the same datasets, we evaluated the consistency scores between the low-input samples and the high-input samples, i.e. the proportions of the REOs of the gene pairs in the high-input RNA samples maintained in the low-input samples. All genes from the gene expression profiles were involved in the REO gene pairs.

**Fig. 3:**
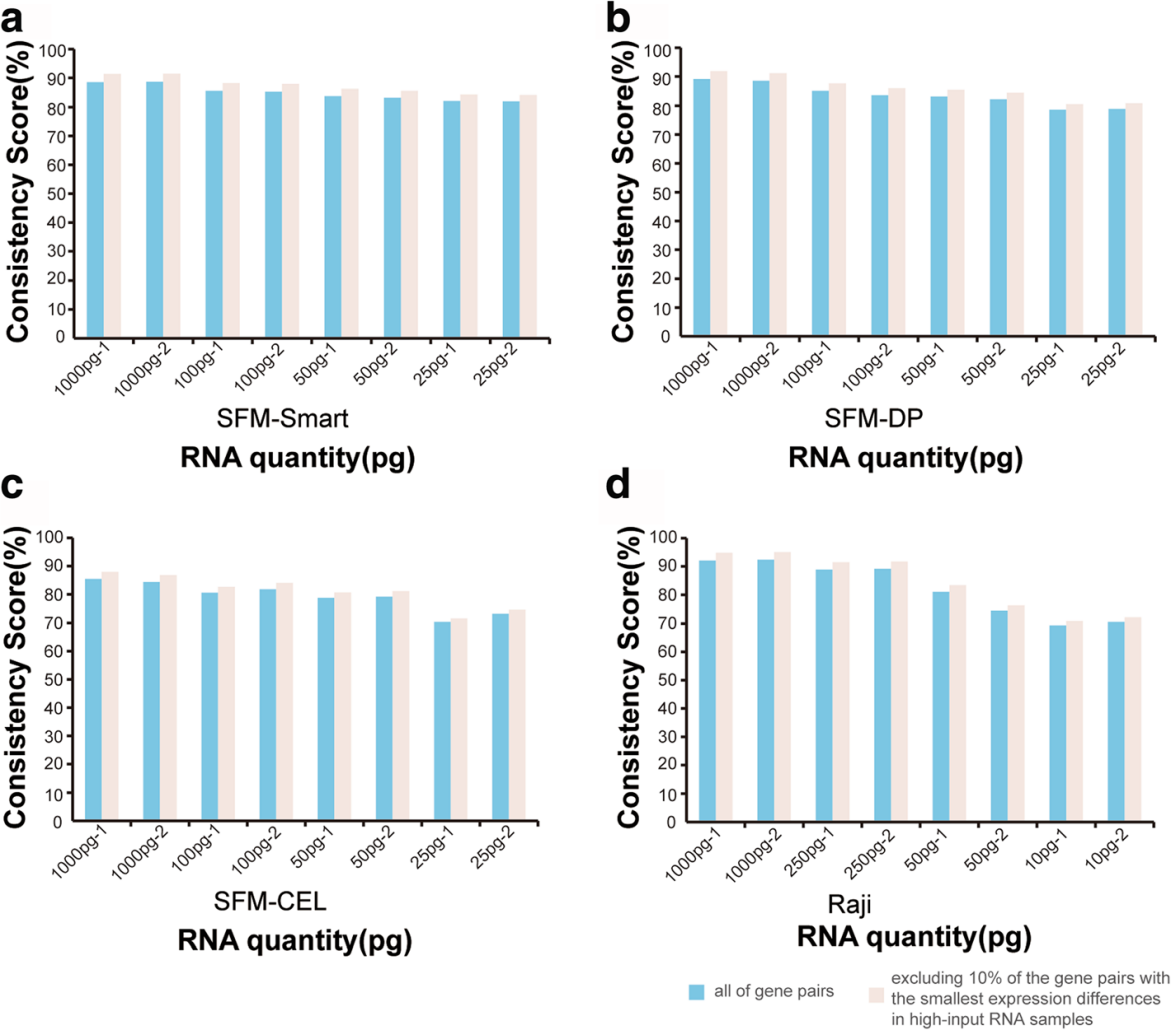
Maintenance of REOs after excluding 0% to 10% gene pairs (**a**) The consistency scores between high-input RNA samples and low-input RNA samples of all gene pairs (blue) and after excluding 10% of the pairs with the smallest expression differences in the paired high-input RNA samples (pink) in the group of SFM-Smart (**b**) (**c**) (**d**) Similar as the Fig. a

In the SFM-Smart dataset GSE50856, 88.53 and 88.63% of the stable REOs in the high-input mRNA samples were respectively kept in the two 1000 pg input mRNA technical replicates. Obviously, the REOs of gene pairs with small rank differences (i.e., close expression levels) tend to be sensitive to random measurement variations [34]. After excluding 10% of pairs with the smallest rank differences, the percentages increased to 91.36 and 91.46% in the two 1000 pg input mRNA technical replicates, respectively. The percentage of the stable REOs in the high-input samples that were kept in the low input technical replicates, termed the consistency scores for short, decreased gradually when the input mRNA decreased. The consistency scores for the two 100 pg input technical replicates decreased to 85.66 and 85.36%, respectively, and increased to 88.24 and 87.92% after excluding the bottom 10% of the gene pairs in the high-input mRNA samples. For the two 50 pg input technical replicates, the consistency scores were 83.84 and 83.29%, respectively, and increased to 86.27 and 85.67%, respectively, after excluding the bottom 10% of the stable gene pairs. For the two 25 pg input technical replicates, the consistency scores were 82.11 and 81.99%, respectively, and increased to 84.39 and 84.26% after excluding the bottom 10% of the gene pairs (Fig. 3a). Similar results were also found in the SFM-DP (Fig. 3b), SFM-CEL (Fig. 3c), AA100-Smart (Additional file 1: Figure S2a), AA100-DP (Additional file 1: Figure S2b) and AA100-CEL groups (Additional file 1: Figure S2c). As shown in the (Additional file 1: Figure S3, Figure S4), the percentage of the stable REOs in the high-input samples that were kept in each of the low input technical replicates increased when more gene pairs with small rank differences in the high-input samples were excluded.

For all the 164,238,402 gene pairs which had the same REOs among two technical replicates of the high-input samples in the Raji dataset GSE17565 measured by the Illumina HumanRef-8 v3.0 platform, 92.09 and 92.43% were respectively kept in the two technical replicates with 1000 pg input total RNA. The consistency scores increased to 94.83 and 95.08%, respectively, after excluding the bottom 10% gene pairs with the smallest rank differences. For the two 250 pg input technical replicates, the consistency scores for all the stable gene pairs were 88.87 and 89.20%, respectively, and increased to 91.49 and 91.76% after excluding the bottom 10% of the gene pairs. For the two 50 pg input technical replicates, the consistency scores were 81.06 and 74.52%, respectively, and increased to 83.39 and 76.42% after excluding the bottom 10% of the gene pairs. For the two 10 pg input technical replicates, the consistency scores were 69.32 and 70.58%, respectively, and increased to 70.87 and 72.23% after excluding the bottom 10% of the gene pairs (Fig. 3d). Similar results were also found in the MCF-7 group (Additional file 1: Figure S4d).

Taken together, the above results showed that the REOs of gene pairs were robust against the amplification bias for the 1000 pg and 250 pg input samples but no longer stable in samples with less than 250 pg RNA input to a certain degree.

### Performance of REOs-based signatures in low-input RNA samples

As a proof of principle that REOs-based signature identified from high-input RNA tissue samples are robust in low-input RNA samples, we collected 69 high-input RNA samples of lymphoma tissues from the GSE55267 dataset and 54 high-input RNA samples of breast cancer tissues from the GSE29431 dataset to search a REOs-based signature for discriminating the two types of tissues (Table 1). We obtained 106,213 highly stable gene pairs that have the same REOs in all lymphoma tissue samples and breast cancer tissue samples, respectively, but the REO patterns were reversal between the two tissue types. From these 106,213 gene pairs, we selected 3 gene pairs (Table 2) with the largest geometric mean of the average absolute rank difference in the lymphoma tissue samples and the average absolute rank difference in the breast cancer tissues samples (see Materials and Methods). The results showed that when *k* = 3 both sensitivity and specificity were 100%. Thus, these three gene pairs with the highest *R*_*ij*_ values, as described in Table 2, were selected as the classification signature. Using the 3 gene pairs as signature, we classified a given sample according to the majority vote rule. If 2 or 3 REOs of the 3 gene pairs in a sample were consistent with the REO patterns in the lymphoma tissue samples, the sample was identified as a lymphoma tissue sample; otherwise, the sample was identified as a breast cancer tissue sample. In the training datasets, obviously, all of the lymphoma tissues samples and the breast cancer tissue samples were correctly classified using the signature. In the independent validation dataset, consisting of 81 high-input RNA samples of lymphoma tissue from the GSE53820 dataset and 30 high-input RNA samples of breast cancer tissue from the GSE10780 dataset, all of the samples were correctly classified.

**Table 1:** High-input RNA tissue samples used in this study

Tissue Sample Type	GEO ID	Sample Size
Lymphoma	GSE55267	69
Breast cancer	GSE29431	54
Lymphoma	GSE53820	81
Breast cancer	GSE10780	30
COAD	TCGA	41
Normal tissues paired with COAD	TCGA	41
Colon tumor tissues	GSE10950	25
Colon normal tissues	GSE10950	25
Colorectal tumors (CRC)	GSE81861	272
Normal mucosas paired with CRC	GSE81861	157

**Table 2:** The 3 gene-pair signature

Gene pair No.	Gene A^a^	Gene B^a^
1	MMP3	RGS13
2	EPCAM	CD37
3	EPCAM	STAP1

^a^Gene A had a higher expression level than Gene B in breast cancer tissues and MCF-7 cell lines

We further applied the REOs-based signature to distinguish Raji and MCF-7 cell lines profiled with high-input with 100 ng total RNA and low-input samples with as low as 50 pg total RNA from the GSE17565 dataset. All the 8 high-input Raji cell line samples, 8 high-input MCF-7 cell line samples, 12 low-input Raji cell line samples and 12 low-input MCF-7 cell line samples were correctly classified. This case study demonstrates that a REOs-based transcriptional signature identified from the high-input RNA tissue samples can be applied to classify low-input samples robustly.

As a second case study, we identified a REO-based signature from high-input RNA samples for discriminating primary colorectal tumors from normal colorectal tissues and showed that it can be robustly applied to low-input RNA samples summarized from single-cell RNA-seq data. Firstly, using the 41 colon adenocarcinoma samples and paired normal samples from TCGA, we identified two lists of gene pairs, each with identical REOs in all samples of the primary colorectal tumor tissue and the corresponding normal tissue, respectively. From the above two lists of gene pairs, 20,390 gene pairs were found to have reversal REOs between the tumor tissues and the normal tissues. Because there were an abundance of dropout events that led to zero expression values for approximately 90% of the genes measured in the single-cell data, it would be inappropriate to select only a few gene pairs as the diagnostic signature. Therefore, all the reversal gene pairs were directly used as the signature. In the training dataset, the 20,390 gene pairs correctly classified all the cancer and normal samples according to the majority voting rule. Then, we collected an independent dataset from GSE10950 with 25 high-input RNA samples of paired colon tumor tissues and colon normal tissues to validate this signature. Because only 18,227 gene pairs of the 20,390 gene pairs were measured in this dataset by the Illumina human Ref-8 v2.0 platform, these 18,227 gene pairs were used to classify the samples according to the majority voting rule and all the samples were correctly classified. However, with the same strategy, 272 colorectal tumor epithelial cells and 157 normal epithelial cells from the GSE81861 dataset could not be correctly classified. This result is not surprising since a cell contains only approximately 10 pg RNA and 90% of genes were measured with zero expression values. The REOs of gene pairs in such small input RNA samples would be unstable as demonstrated above. To address this issue, we constructed a pooled dataset from the single-cell RNA-seq results.

In the GSE81861 dataset, the 272 tumor epithelial cells and the 157 normal epithelial cells were extracted from 11 patients of primary colorectal tumors and paired normal tissues; however, there were no annotation on patients’ information. We randomly assigned the 272 colorectal tumor epithelial cells into 11 samples with approximately equal number of cells: 10 samples each with 25 single cells and a sample with 22 single cells. Each simulated disease sample contains approximately 250 pg RNA. Similarly, the 157 normal epithelial cells were also randomly assigned into 10 samples each with 14 single cells and a sample with 17 single cells. Each sample approximately contains 140 pg RNA. In each sample, we calculated the sum of the measurement values for each gene to represent the expression levels of the genes [19]. Then, the REO signature with 20,390 gene pairs constructed from the high-input RNA samples was applied to classify the simulated low-input RNA samples from the single-cell data. Because only 18,308 gene pairs of the 20,390 gene pairs were measured in single cells by the Illumina HiSeq 2000 platform, we used the measured 18,308 gene pairs to classify the samples according to the majority voting rule. This random experiment was repeated for 100 times. The results showed that the average sensitivity and specificity were 100 and 73.55%, respectively. As demonstrated in the above Section, the REOs of gene pairs in the input samples with less than 250 pg RNA input tends to be less robust against the amplification bias. Therefore, for the 18,308 gene pairs, we respectively excluded 10 and 20% of pairs with the smallest average rank differences in either the normal samples or the disease samples, and used the remained gene pairs to classify the samples. For 100 random experiments, while the average sensitivity was kept at 100%, the average specificity increased to 91.82% (or 100%) when 10% (or 20%) of the gene pairs with the smallest average rank differences in either the normal samples or the disease samples were excluded.

## Discussion

It is crucial to develop reliable analysis methods for the precise monitoring of global gene expression levels in limited clinical tissues in many research areas of biological and medical disciplines. For those methods based on quantitative gene expression values such as differential genes and risk score signatures, there exists large uncertainty for the low-input RNA samples due to inherent amplification bias and technical noise in the amplification procedures. However, the relative expression orderings of gene pairs are tolerant to these issues, which suggests us that we should take the advantage of the robustness of REOs to gain more reliable biological insight.

We compared serially diluted RNA samples to evaluate the impact of amplification techniques for low-input RNA samples on the gene expression profile measurements. As displayed in the study, thousands of genes had at least 2 folds-change of expression measurements in the low-input RNA samples compared with the paired high-input RNA samples due to the amplification procedure. Consequently, for the transcriptional signatures based on the quantitative expression levels, the risk threshold values determined from high-input RNA samples could not be applied to low-input RNA samples directly and vice versa. In contrast, we found that approximately 90% of REOs of gene pairs in high-input RNA samples were maintained in the diluted 1000 pg, low-input mRNA samples which were amplified and profiled by Smart-seq, DP-seq and CEL-seq techniques using the Illumina HiSeq 2000 platform. For the low-input total samples which were amplified and profiled by the Whole-Genome DASL technique using the Illumina HumanRef-8 v3.0 platform, at least 90% of REOs of gene pairs in the high-input samples were maintained in the diluted 1000 pg and 250 pg input samples but unstable in the 50 pg and10 pg input samples to a certain degree.

Our REO-based method facilitates gene expression profiling analysis in the context where the starting RNA material is extremely limited. A problem with the current study is that we cannot find appropriate data to verify the clinical value of the REOs-based signature. For the future study, it is worthwhile to further evaluate the method using clinically meaningful low-input RNA data such like tissues from minimally invasive tissue biopsy techniques and single-cell samples.

## Conclusions

Thousands of genes have at least 2 folds-change of expression measurements in low-input mRNA and total RNA samples compared with the corresponding paired high-input samples. In contrast, most of the REOs of gene pairs in the high-input samples are maintained in the diluted low-input samples. Therefore, REOs-based disease signatures determined from high-input samples can be robustly applied to low-input samples.

## Methods

### Data sources and data preprocessing

All the gene expression data analyzed in this study were downloaded from the GEO database (http://www.ncbi.nlm.nih.gov/geo/), as described in details in Fig. 1 and Table 1. In Fig. 1, there are 2 datasets including mRNA sequencing data and whole genome gene expression data which were used to evaluate the amplification bias. In Table 1, there are, in total, 6 datasets of high-input RNA tissue samples, including 4 sets which were used to obtain the classification signature between breast cancer and lymphoma cancer and 2 datasets which were used to obtain the classification signature between colon tumor tissues and normal tissues.

The gene expression profiles of dataset GSE50856 were measured by the Illumina HiSeq 2000 platform for the low-input mRNA samples collected from day-4 embroid bodies of mouse embryonic stem cells (mESCs) differentiated in serum free media with and without Activin A treatment. The control samples were labeled with “SFM” and the Activin A-treated samples were labeled with “AA1000”. The low-input samples were amplified and profiled by Smart-seq, DP-seq and CEL-seq techniques using the Illumina HiSeq 2000 platform, with those of the paired high-input (50 ng) mRNA samples. Based on the amplification methods and cell line treatment status, the dataset was divided into 6 groups: SFM-Smart, SFM-DP, SFM-CEL, AA100-Smart, AA100-DP and AA100-CEL. Each group had four input levels, 1000 pg, 100 pg, 50 pg and 25 pg, and each level had two technical replicates. The gene expression profile of dataset GSE17565 was measured by the Illumina HumanRef-8 v3.0 platform for two cell lines, Raji and MCF-7. The dataset was divided into 2 groups: Raji and MCF-7. The low-input total RNA samples were amplified and profiled by the Whole-Genome DASL technique using the Illumina HumanRef-8 v3.0 platform using the Illumina HumanRef-8 v3.0 platform, with those of the paired high-input (100 ng) total RNA samples. There were four input levels, 1000 pg, 100,250 pg, 50 pg and 25 10 pg as well for both cell lines, and every input level had two technical replicates (Fig. 1).

For the GSE50856 dataset, we downloaded the mappable reads that fell onto gene’s exons. The experiments of standard RNA-seq, Smart-seq and DP-seq are single-end RNA-seq where every read corresponds to a single fragment. Thus, the RPKM (reads per kilobase of exon model per million mapped reads) and FPKM (fragments per kilobase of exon model per million mapped reads) metrics are conceptually analogous [35, 36], which could be used to quantify the gene expression level. The RPKM metric was estimated by the formula [37]: R = (10^9*C)/NL, where C is the number of mapped reads that fell onto the gene’s exons, N is the total number of mapped reads in the experiment, and L is the sum of the exons in base pairs. On the other hand, The experiment of CEL-seq is paired-end sequencing where two reads correspond to a single fragment and only FPKM could be used to quantify the gene expression level. For the paired-end experiment, the FPKM value would be half of the RPKM value. This is not always true because in some cases only one of the two reads belonging to a fragment might be mapped. However, for most applications this simplification works [35]. The mouse mm9 genome was used for the genome annotation. By transforming the gene bank accession ID providing in the GSE50856 dataset into Entrez gene ID through the Source Batch Search database (http://source-search.princeton.edu/cgi-bin/source/sourceBatchSearch), 20,541 genes were analyzed in this dataset.

For dataset GSE17565 measured by Illumina HumanRef-8 v3.0 platform, dataset GSE10950 measured by Illumina humanRef-8 v2.0 platform and dataset GSE81861 measured by Illumina HiSeq 2000 platform, we directly downloaded the processed data. For 4 datasets of the expression profiles measured by Affymatrix microarrays, the raw expression data (.CEL files) were preprocessed using the Robust Multi-array Average algorithm [38]. For the data from TCGA, the level 3 RNA-seq datasets (RNAseqV2 RSEM) of mRNA were downloaded from the Broad Institute, Firehose (http://gdac.broadinstitute.org/runs/stddata__2016_01_28/).

### Evaluation on amplification bias by fold change

For each of the measured genes, we calculated the average of its expression values in the technical replicates for the low input and the paired high input RNA samples, respectively, and then calculated the fold changes (FCs) between the low input RNA samples and the paired high input RNA samples. We also calculated the FC between every paired low input RNA technical replicate and high input RNA technical replicates, and then calculated the coefficient of variation (CV) of the FCs.

### Evaluation on REOs of gene pairs

Highly stable REOs of the gene pairs were obtained respectively from high-input RNA samples and low input RNA samples. We defined a REO as highly stable if the gene pair had identical REO direction in both technical replicates of one sample. The details are as following. The comparison of two genes in a gene pair (*G*_*i*_, *G*_*j*_) was viewed as an event with only two possible outcomes: the expression level of *G*_*i*_ was either higher or lower than that of *G*_*j*_ and the relative expression ordering was denoted as *G*_*i*_ > *G*_*j*_ or *G*_*i*_ < *G*_*j*_. If the REO of a pair was maintained in more than 99% of samples, the pair was called a highly stable gene pair. The REOs of two genes with small rank difference (i.e., close expression levels) tend to be unstable due to measurement variations.

To compare two lists of stable REOs, the consistency score, which was defined as *k*/*n*, was calculated, where *n* was the number of the gene pairs in the high-input RNA samples and *k* was the number of gene pairs with the consistent REOs in both the high-input RNA samples and low-input RNA samples.

### REOs-based signature from high-input RNA samples for discriminating cancer tissues

First, we identified gene pairs each with identical REO in all samples of the two types of tissues, respectively, but with reversal REO patterns between the two types of samples. Then, we calculated the reversal degree for each gene pair as following equation,

$$ \overline{\text{\textsf{\textit{R}}}}_{\text{\textsf{\textit{ij}}}} = \sqrt{\overline{\text{\textsf{\textit{R}}}}_{\text{\textsf{\textit{ij}}}(\text{\textsf{{lym}}})}}\ \overline{\text{\textsf{\textit{R}}}}_{\text{\textsf{\textit{ij}}}(\text{\textsf{{bre}}})} $$

where‾*R*_*ij*(lym)_ and‾*R*_ij(bre)_ are the arithmetic means of the absolute rank differences of the gene pair (*i*, *j*) in all samples of the two types of tissues, respectively.

Second, the gene pairs with reversal REOs were sorted in a descending order according to their reversal degrees. Obviously, the larger the *R*_*ij*_ value, the larger the reversal degree of the REO is between the two types of samples. Third, we selected the top *k* gene pairs, where *k* is an odd integer ranging from 1 to the total number of candidate gene pairs to classify the samples based on the majority vote rule. The value of *k* was chosen as the smallest number of gene pairs that reached the highest geometric mean of the sensitivity and specificity in the classification tests. Then, the selected gene-pair signature was tested in independent tissue samples measured with high-input RNA and in the cell line data measured with low-input RNA.

### Performance evaluation

We called lymphoma tissue samples, colorectal tumor epithelial cells as positive samples, breast cancer tissue samples, Normal mucosa’s epithelial cells paired with colorectal tumor as negative samples, and evaluated the performance of the classification signature using sensitivity and specificity which are calculated as follows:

$$ \mathrm{Sensivity}=\frac{TP}{TP+ FN} $$

$$ \mathrm{Specificity}=\frac{TN}{TN+ FP} $$

where TP, TN, FP and FN denote the number of true positives, true negatives, false positives and false negatives, respectively.

### Statistical software for analysis

All statistical analyses were performed using the R 3.1.3 (http://www.r-project.org/). The main analyses codes are provided in the (Additional file 2).

## Additional files

Additional file 1: Figure S1. The coefficient of variation (CV) of FCs. **(a)** The coefficient of variation (CV) of FCs in the three groups of SFM-DP, SFM-CEL and SFM-Smart respectively in the 25 pg, 50 pg, 100 pg and 1000 pg RNA quantity **(b) (c) **Similar as the Figure a. **Figure S2.** Maintenance of REOs after excluding 0 to 10% gene pairs. **(a)** The consistency scores between high-input RNA samples and low-input RNA samples of all gene pairs (blue) and after excluding 10% of the pairs with the smallest expression differences in the paired high-input RNA samples (pink) in the group of AA100-Smart **(b) (c) (d)** Similar as the Figure a. **Figure S3.** Maintenance of REOs after excluding 0 to 30% gene pairs. The consistency scores between high-input RNA samples and low-input RNA samples of all gene pairs, after excluding 0, 5, 10, 15, 20 and 30% of the pairs with the smallest expression differences in the paired high-input RNA samples in the group of AA100-Smart **(b) (c) (d)** Similar as the Figure a. (PDF 606 kb)

Additional file 2: The main analyses codes used in this research. (R 4 kb)

## Abbreviations

AA100: Activin A treatment
AA100-CEL: Mouse embryonic stem cells differentiated in control serum free media (SFM) and the collected RNA amplified and profiled by CEL-seq using the Illumina HiSeq 2000 platform
AA100-DP: Mouse embryonic stem cells differentiated in control serum free media (SFM) and the collected RNA amplified and profiled by DP-seq using the Illumina HiSeq 2000 platform
AA100-Smart: Mouse embryonic stem cells subjected to Activin A treatment (AA100) and the collected RNA amplified and profiled by Smart-seq using the Illumina HiSeq 2000 platform
CEL-seq: Cell expression by linear amplification and sequencing
COAD: Colon adenocarcinoma
CRC: Colorectal tumors
DP-seq: Designed Primer-based RNA-sequencing strategy
FC: Fold change
GEO: Gene Expression Omnibus
REOs: Within-sample relative expression orderings
SFM: Serum free media
SFM-CEL: Mouse embryonic stem cells differentiated in control serum free media (SFM) and the collected RNA amplified and profiled by CEL-seq using the Illumina HiSeq 2000 platform
SFM-DP: Mouse embryonic stem cells differentiated in control serum free media (SFM) and the collected RNA amplified and profiled by DP-seq using the Illumina HiSeq 2000 platform
SFM-Smart: Mouse embryonic stem cells differentiated in control serum free media (SFM) and the collected RNA were amplified and profiled by Smart-seq using the Illumina HiSeq 2000 platform
SMART: Switching mechanism at 5′-end of the RNA transcript
