# Supplementary material for: Robust transcriptional signatures for low-input RNA samples based on relative expression orderings
Source: BMC Genomics. 2017 Nov 28;18:913. doi: 10.1186/s12864-017-4280-7 (PMC5704640; doi:10.1186/s12864-017-4280-7)

Supplementary Figure 1. The coefficient of variation (CV) of FCs

(a) The coefficient of variation (CV) of FCs in the three groups of SFM-DP, SFM-CEL and SFM-Smart respectively in the 25 pg, 50pg, 100pg and 1000pg RNA quantity **(b) (c)**Similar as the Figure a

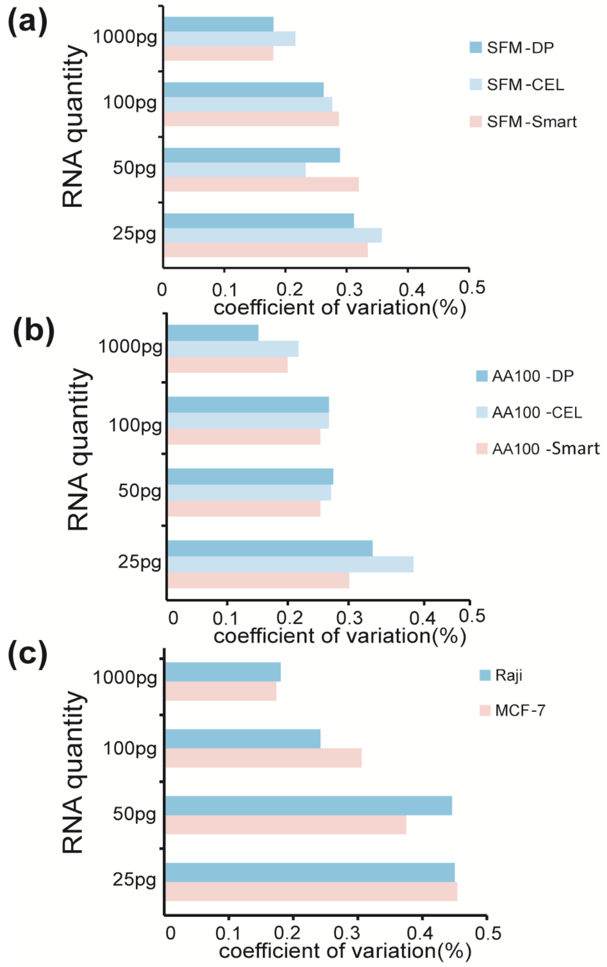

Supplementary Figure 2. Maintenance of REOs after excluding 0% to 10% gene pairs

(a) The consistency scores between high-input RNA samples and low-input RNA samples of all gene pairs (blue) and after excluding 10% of the pairs with the smallest expression differences in the paired high-input RNA samples (pink) in the group of AA100-Smart **(b)** **(c)** **(d)** Similar as the Figure a

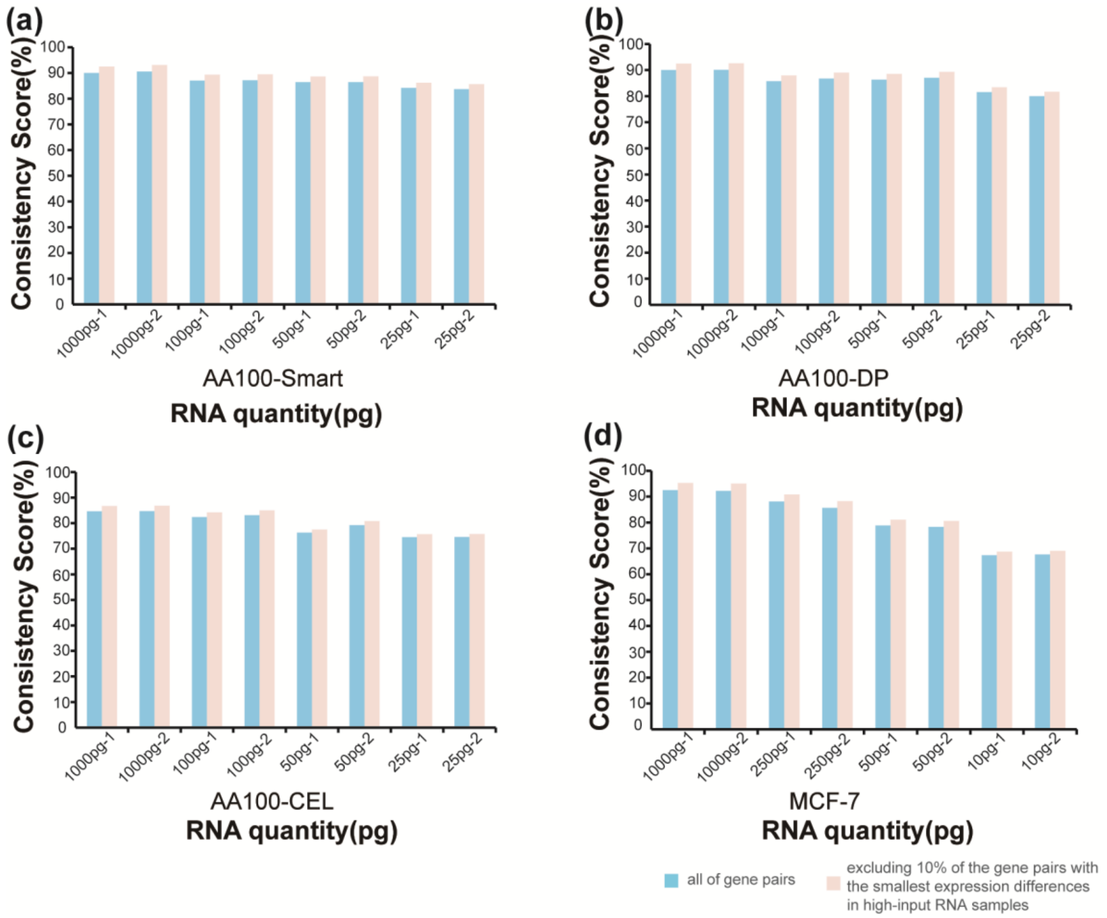

Supplementary Figure 3. Maintenance of REOs after excluding 0% to 30% gene pairs

(a) The consistency scores between high-input RNA samples and low-input RNA samples of all gene pairs, after excluding 0%, 5%, 10%, 15%, 20% and 30% of the pairs with the smallest expression differences in the paired high-input RNA samples in the group of AA100-Smart **(b) (c) (d)** Similar as the Figure a

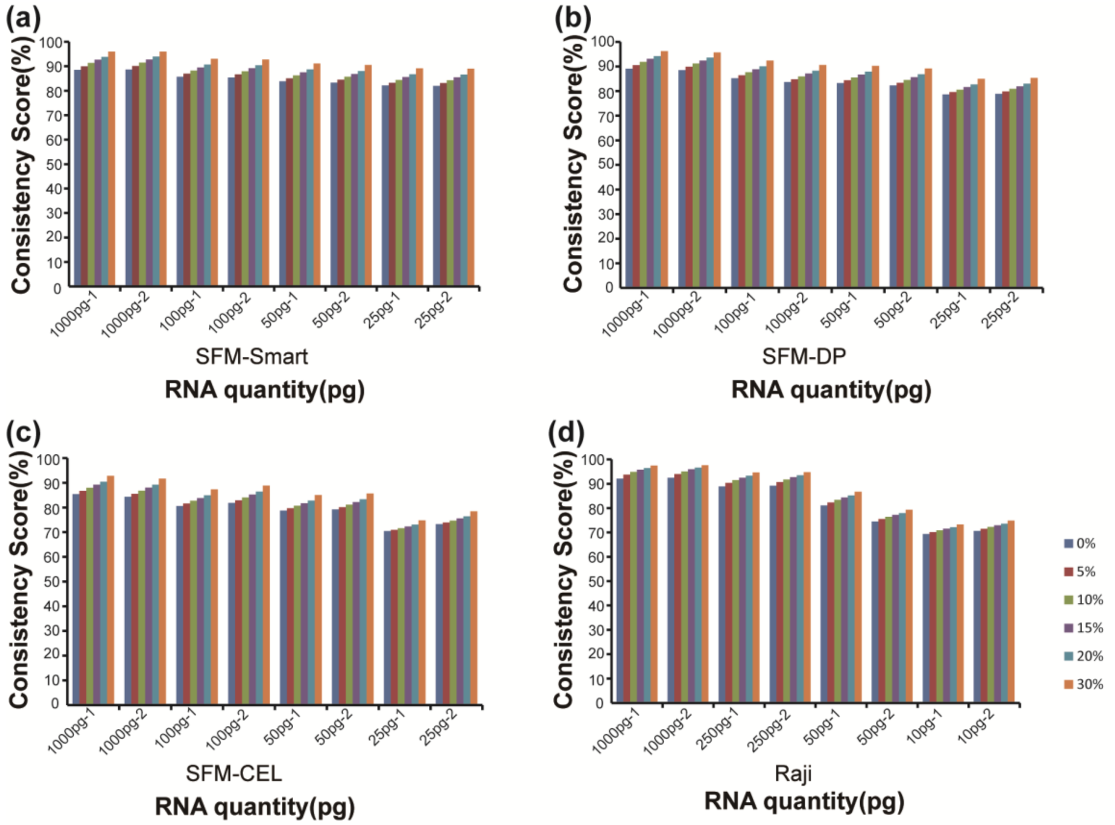

Supplementary Figure 4. Maintenance of REOs after excluding 0% to 30% gene pairs

(a) The consistency scores between high-input RNA samples and low-input RNA samples of all gene pairs, after excluding 5%, 10%, 15%, 20% and 30% of the pairs with the smallest expression differences in the paired high-input RNA samples **(b) (c) (d)** Similar as the Figure a

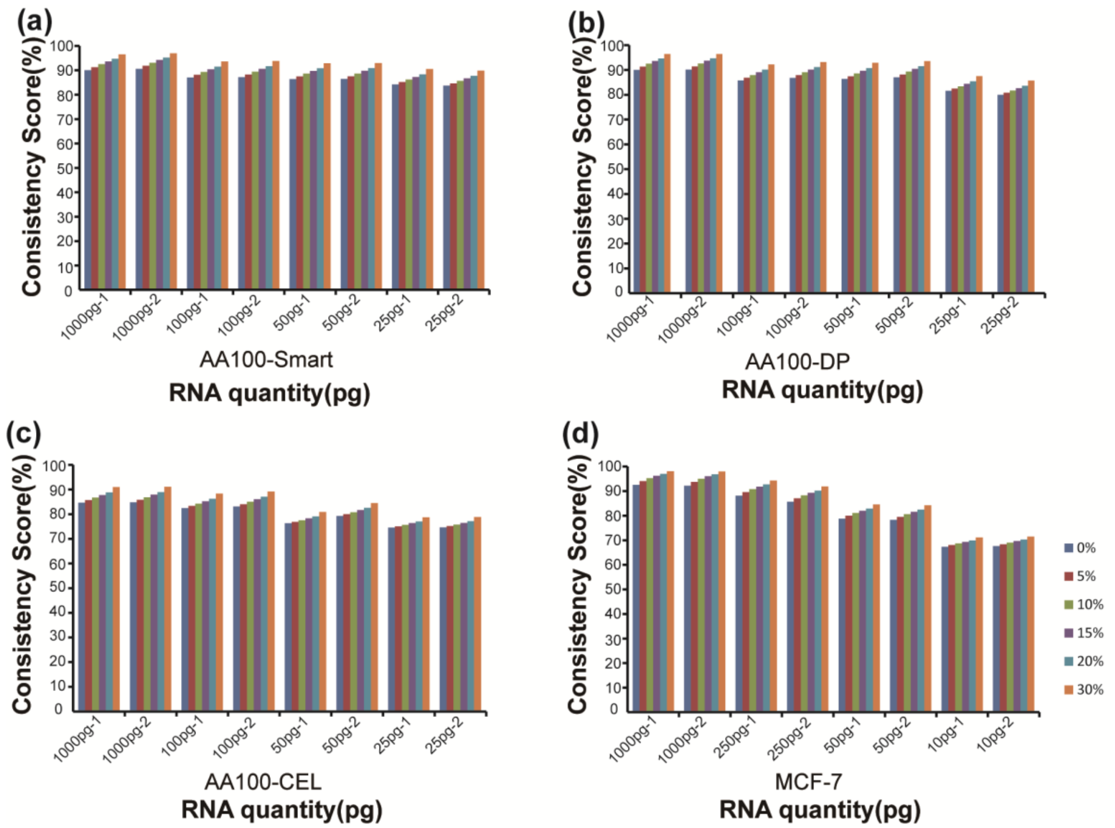

Supplement: Supplementary file 1 — The coefficient of variation (CV) of FCs. (a) The coefficient of variation (CV) of FCs in the three groups of SFM-DP, SFM-CEL and SFM-Smart respectively in the 25 pg, 50 pg, 100 pg and 1000 pg RNA quantity (b) (c) Similar as the Figure a. Figure S2. Maintenance of REOs after excluding 0 to 10% gene pairs. (a) The consistency scores between high-input RNA samples and low-input RNA samples of all gene pairs (blue) and after excluding 10% of the pairs with the smallest expression differences in the paired high-input RNA samples (pink) in the group of AA100-Smart (b) (c) (d) Similar as the Figure a. Figure S3. Maintenance of REOs after excluding 0 to 30% gene pairs. The consistency scores between high-input RNA samples and low-input RNA samples of all gene pairs, after excluding 0, 5, 10, 15, 20 and 30% of the pairs with the smallest expression differences in the paired high-input RNA samples in the group of AA100-Smart (b) (c) (d) Similar as the Figure a. (PDF 606 kb) [file 12864_2017_4280_MOESM1_ESM.pdf]
